# Insights into Broilers' Gut Microbiota Fed with Phosphorus, Calcium, and Phytase Supplemented Diets

**DOI:** 10.3389/fmicb.2016.02033

**Published:** 2016-12-19

**Authors:** Daniel Borda-Molina, Marius Vital, Vera Sommerfeld, Markus Rodehutscord, Amélia Camarinha-Silva

**Affiliations:** ^1^Animal Nutrition Department, Institute of Animal Science, University of HohenheimStuttgart, Germany; ^2^Microbial Interactions and Processes Research Group, Helmholtz Centre for Infection ResearchBraunschweig, Germany

**Keywords:** 16S sequencing, microbiota, chicken GIT, digesta, mucosa, phosphorus, calcium, phytase

## Abstract

Phytase supplementation in broiler diets is a common practice to improve phosphorus (P) availability and to reduce P loss by excretion. An enhanced P availability, and its concomitant supplementation with calcium (Ca), can affect the structure of the microbial community in the digestive tract of broiler chickens. Here, we aim to distinguish the effects of mineral P, Ca, and phytase on the composition of microbial communities present in the content and the mucosa layer of the gastrointestinal tract (GIT) of broiler chickens.

Significant differences were observed between digesta and mucosa samples for the GIT sections studied (*p* = 0.001). The analyses of 56 individual birds showed a high microbial composition variability within the replicates of the same diet. The average similarity within replicates of digesta and mucosa samples across all diets ranged from 29 to 82% in crop, 19–49% in ileum, and 17–39% in caeca. Broilers fed with a diet only supplemented with Ca had the lowest body weight gain and feed conversion values while diets supplemented with P showed the best performance results. An effect of each diet on crop mucosa samples was observed, however, similar results were not obtained from digesta samples. Microbial communities colonizing the ileum mucosa samples were affected by P supplementation. Caeca-derived samples showed the highest microbial diversity when compared to the other GIT sections and the most prominent phylotypes were related to genus *Faecalibacterium* and *Pseudoflavonifractor*, known for their influence on gut health and as butyrate producers. Lower microbial diversity in crop digesta was linked to lower growth performance of birds fed with a diet only supplemented with Ca. Each diet affected microbial communities within individual sections, however, no diet showed a comprehensive effect across all GIT sections, which can primarily be attributed to the great variability among replicates. The substantial community differences between digesta and mucosa derived samples indicate that both habitats have to be considered when the influence of diet on the gut microbiota, broiler growth performance, and animal health is investigated.

## Introduction

Broiler chickens are one of the most used farm animals due to the efficient conversion of feed into body weight gain (Stanley et al., 2014). Phosphorus (P) supply with the diet plays an important role in skeletal system development and maintenance of chickens. P is, however, a non-renewable resource that is expected to be depleted in the next 100 years (Shastak and Rodehutscord, 2013). Phytate, an organic source of P contained in plant seeds and plant-based diets for broilers, is a principal source of P for the animal, but it has the disadvantage of not being easily accessible by broilers (Witzig et al., 2015; Zeller et al., 2015). The P availability of plant-based diets can be improved by supplementing the diets with phytase, an enzyme that increases P digestibility and reduces P excretion (Witzig et al., 2015). In consequence, the amount of calcium (Ca) and P required in diet formulation can be reduced following release of these two elements from phytate complexes (Zeller et al., 2015). Changes in Ca and P supplementation affected the composition and activity of the microbial community in the digestive tract of broilers (Ptak et al., 2015). Because the microbes are involved to a variable extent in enzymatic hydrolysis of nutrient fractions in the digestive tract, it is necessary to understand the role of the microbial community of the gut (Eeckhaut et al., 2011) and its interaction with the host, in order improve the utilization of nutrients such as phytate bound P by the bird.

The microbial community present in the broilers' gastrointestinal tract (GIT) has more than 900 bacterial species (Stanley et al., 2014). They play a crucial role in feed digestion, breakdown of toxins, exclusion of pathogens, stimulation of the immune system, and endocrine activity (Zhu et al., 2002). Several studies have analyzed the microbiota from specific sections of the GIT including the crop, ileum, and caeca (Sekelja et al., 2012; Sergeant et al., 2014; Ptak et al., 2015; Witzig et al., 2015), whereas only a few have focused on the whole GIT (Lu et al., 2003; Sekelja et al., 2012). Nonetheless, it is now known that they are highly connected and should influence up and down-stream the different GIT sections (Stanley et al., 2014). Most studies have focused on content of the GIT (digesta) samples only (Sekelja et al., 2012; Walugembe et al., 2015; Witzig et al., 2015), ignoring the mucosa communities, that are the closest to the host epithelium (Collado and Sanz, 2007). Epithelium attached microbial communities have biological roles that should be characterized. A high bacterial diversity was observed in the *Pars non glandularis* of the pig stomach (Mann et al., 2014) and previous reports in rats and humans have found differences between the microbial counts in the colonic mucosa and feces (Zoetendal et al., 2002; Haange et al., 2012).

The crop, the section where feed is temporally stored and fermentation activities initiate, is highly dominated by *Lactobacillus* species (Stanley et al., 2014; Witzig et al., 2015). The ileum, where nutrients are absorbed, is mainly colonized by *Lactobacillus* species and also by partially characterized bacteria with butyrate producing activities, such as *Clostridium, Streptococcus*, and *Enterococcus* (Stanley et al., 2014). The caeca, where complex substrates such as cellulose, other polysaccharides, and phytate are fermented (Stanley et al., 2014; Choi et al., 2015; Zeller et al., 2015) is the most diverse section of the GIT and is highly dominated by unknown microbes. The most abundant families in caeca are Clostridiaceae, Bacteroidaceae, Lactobacillaceae, and butyrate producers (Stanley et al., 2014).

Considering the low availability of P in plant-based diets, and the effect of supplementing diets with phytase, Ca, and P on chickens' performance and phytate degradation in the digestive tract, this study aims to investigate the influence of these supplements, on the microbial communities of digesta and mucosa samples of three sections of the GIT of broiler chickens.

## Materials and methods

### Animal sampling

The animal experiment was carried out in the Agricultural Experiment Station of Hohenheim University, location Lindenhöfe in Eningen (Germany). All procedures regarding animal handling and treatments were approved by the Regierungspräsidium Tübingen (approval number HOH33|14TE).

A total of 1064 broiler chickens (unsexed, strain Ross 308) were allocated to 56 floor pens. Animals were fed with a commercial starter diet (Table S1) until day 14 of age. On day 15 each pen was randomly assigned to one of eight different dietary treatments (seven pens per diet; Table 1). The diets were mixed based on corn and soybean meal (Table S1) with the supplementation of two levels of P (monosodium phosphate; 0 or 2 g P/kg), Ca (limestone; 0 or 3 g Ca/kg), and an *E. coli*-derived 6-phytase Quantum™ Blue, AB Vista (0 or 1500 FTU/kg; Table 1). The experiment followed a 2 × 2 × 2 factorial arrangement of treatments. On day 26 one animal per pen was euthanized by carbon dioxide asphyxiation following anesthesia in a gas mixture (35% CO_2_, 35% N_2_, and 30% O_2_; Zeller et al., 2015). The GIT was dissected immediately after euthanization and crop, ileum (terminal two-thirds of the section between Meckel's diverticulum and 2 cm anterior to the ileo-ceco-colonic junction) and the two caeca, were opened longitudinally and digesta samples were collected with a sterile spoon. The mucosa was washed with sterile phosphate-buffered saline and scraped with a sterile glass slide. In some cases, the amount of digesta contained in a certain section was not sufficient, resulting in a total of 281 samples collected, which included 3–7 replicates per dietary treatment and sample type (mucosa and digesta; Table S2A). Samples were stored at −80°C.

**Table 1:** **Phosphorus (P), calcium (Ca), and phytase concentration in the eight dietary treatments**.

**Diets**	**A**	**B**	**C**	**D**	**E**	**F**	**G**	**H**
	**P−**	**P−**	**P−**	**P−**	**P+**	**P+**	**P+**	**P+**
	**Ca−**	**Ca−**	**Ca+**	**Ca+**	**Ca−**	**Ca−**	**Ca+**	**Ca+**
	**Ph−**	**Ph+**	**Ph−**	**Ph+**	**Ph−**	**Ph+**	**Ph−**	**Ph+**
Total-P (g/kg)	4.1	4.1	4.1	4.1	6.9	6.9	6.9	6.9
Ca (g/kg)	6.2	6.2	10.4	10.4	6.2	6.2	10.4	10.4
Phytase (FTU/kg)^a^	0	1500	0	1500	0	1500	0	1500

a *The calculated activity in the diet is based on enzyme supplements; intrinsic enzyme activity is not included. −, without supplementation; +, with supplementation*.

### Broiler performance analysis

Information regarding final body weight (BW), feed consumption (FC), BW gain and feed to gain ratio, was obtained from day 15 to 26 and analyzed with MIXED procedure of the software SAS (version 9.1.3, SAS Institute, Cary, NC). The statistical model was y_jjklm_ = μ + r_i_ + T_j_ + β_k_ + x_l_ + (Tβ)_jk_ + (Tx)_jl_ + (βx)_kl_ + (Tβx)_jkl_ + e_ijklm_; where μ = general mean, r_i_ = effect of the block (random), T_j_ = effect of the P addition (fixed), β_k_ = effect of the Ca addition (fixed), x_l_ = effect of the phytase addition (fixed), (Tβ)_jk_, (Tx)_il_, (βx)_kl_ are the two factor interactions, (Tβx)_jkl_ are the three factor interaction and e_ijklm_ = random error of the observations. Statistical significance was evaluated by one-way ANOVA. Differences between treatments were tested with a multiple *t*-test (LSD). A significance level of *p* ≤ 0.05 was considered.

### DNA extraction and illumina amplicon sequencing

DNA was extracted from 281 samples with FastDNA™ SPIN Kit for soil from MP Biomedicals (Solon, OH, USA), following the instructions of the manufacturer's protocol. DNA was quantified in a NanoDrop 2000 spectrophotometer (Thermo Scientific, Waltham, MA, USA) and stored at −20°C.

Illumina library preparation with PCR amplification of the V1-2 region of the 16S rRNA gene using PrimeSTAR HS DNA Polymerase (Clontech Laboratories, Mountain View, CA, USA) was performed according to Camarinha-Silva et al. (2014). Amplicons were verified by agarose gel electrophoresis, purified with Macherey-Nagel 96-well-plate (Macherey Nagel, Düren, Germany) and quantified using a QuantiFluor® dsDNA system (Promega, Madison, USA). Equimolar ratios of amplicons (30 ng) were pooled followed by an ethanol precipitation in order to remove any contaminants. Correct size of the PCR product was obtained and purified with QIAquick gel extraction kit (Qiagen, Hilden, Germany). Libraries were sequenced using 250 bp paired-end sequencing chemistry on an Illumina MiSeq platform.

Bioinformatic processing of sequences was done according to Camarinha-Silva et al. (2014) with some modifications. Raw reads were assembled (Cole et al., 2014) and subsequently aligned using MOTHUR (gotoh algorithm with the SILVA reference database) prior to pre-clustering (diffs = 2). Sequences were clustered into operational taxonomic units (OTU) at ≥97% similarity. All OTUs with an average abundance lower than 0.001% across all the samples and with sequence length <250 bp were discarded from the analysis. Finally, 293,862 ± 1459 sequences were obtained per sample comprising a total of 1796 OTUs that were taxonomically assigned using the naïve Bayesian RDP classifier (Wang et al., 2007; Table S3). OTUs were then manually evaluated against the RDP database using Seqmatch function. Sequences are available at the European Nucleotide Archive (ENA) under accession number PRJEB14628 in http://www.ebi.ac.uk/ena/data/view/PRJEB14628.

### Multivariate analysis

A multivariate dataset with the respective abundances of each OTU on each sample was analyzed using PRIMER (version 7.0.9, PRIMER-E, Plymouth Marine Laboratory, Plymouth, UK; Clarke and Warwick, 2001). Data was standardized and a sample similarity matrix was created using Bray-Curtis coefficient (Bray and Curtis, 1957). The community similarity structure was depicted through non-metric multidimensional scaling plots (nMDS) and shade plots were used to study species distributions between the diets and each section (Clarke and Warwick, 2001). Similarity percentages analysis (SIMPER) identified the species contribution to the Bray-Curtis similarity among samples within each diet (Clarke and Warwick, 2001). PERMANOVA routine was used to study the significant differences and interactions between factors [diet, type of sample (digesta or mucosa) and GIT section], and differences between the diets were studied based on the pair-wise tests using a permutation method under a reduced model. Pielou's evenness index and Shannon-weaver index of diversity (H′) were used to calculate OTUs evenness and diversity.

Differences in the abundance of OTUs of interest between diets were evaluated using the unpaired Welch's *t*-test that can handle unequal variances, unequal sample sizes and non-parametric data (Welch, 1947). OTUs abundances were considered significantly different if *p* < 0.05.

Correlations were estimated with Pearson correlation coefficient (999 permutations) using PRISM 6 (GraphPad Software, CA). Correlations were considered significantly different if *p* < 0.05.

## Results and discussion

### Global overview of broiler performance and the microbial community in crop, ileum, and caeca

The growth performance of broiler chickens was significantly affected by the levels of P, Ca, phytase, and their corresponding interactions (Table 2). Final BW, FC, and BW gain increased in diets that included P supplementation (E, F, G, and H) and in diet B with only phytase supplementation (Tables 1, 2). The growth performance of birds on these diets was significantly different from the others. The lowest performance birds were those on diet C, with only supplementation of Ca

**Table 2:** **Broiler chickens performance data between day 15 and 26 for the eight dietary treatments**.

**Diets**	**A**	**B**	**C**	**D**	**E**	**F**	**G**	**H**
	**P−**	**P−**	**P−**	**P−**	**P+**	**P+**	**P+**	**P+**
	**Ca−**	**Ca−**	**Ca+**	**Ca+**	**Ca−**	**Ca−**	**Ca+**	**Ca+**
	**Ph−**	**Ph+**	**Ph−**	**Ph+**	**Ph−**	**Ph+**	**Ph−**	**Ph+**
Final BW (g)	1433^bc^	1527^a^	1202^d^	1420^c^	1510^a^	1539^a^	1492^ab^	1530^a^
FC (g/d)	117^b^	121^ab^	96^d^	112^c^	124^a^	123^a^	119^ab^	122^a^
BW gain (g/d)	78^b^	86^a^	58^c^	76^b^	86^a^	86^a^	83^a^	86^a^
F:G (g/g)	1.49^b^	1.41^d^	1.66^a^	1.47^bc^	1.44^cd^	1.42^d^	1.44^cd^	1.41^d^
	***p*****-value**							
	**Pooled *SD***	***P***	**Ca**	**Phy**	P^*^Ca	P^*^phy	Ca^*^phy	P^*^Ca^*^phy
Final BW (g)	21.02	< 0.0001	< 0.0001	< 0.0001	< 0.0001	0.0003	0.0383	0.0756
FC (g/d)	1.26	< 0.0001	< 0.0001	< 0.0001	< 0.0001	< 0.0001	0.0006	0.0526
BW gain (g/d)	1.31	< 0.0001	< 0.0001	< 0.0001	< 0.0001	< 0.0001	0.0007	0.0406
F:G (g/g)	0.012	< 0.0001	< 0.0001	< 0.0001	< 0.0001	< 0.0001	0.0178	0.0407

*Final body weight (BW), feed consumption (FC), BW gain and feed to gain (F:G) ratio of broiler chickens. Data are given as treatment means with respective SD (standard deviation); n = 7 blocks, 16–18 animals per block, and means without common superscript resulted being significantly different (p < 0.05)*.

Based on the taxonomic threshold defined by Yarza et al. (2014), which takes into consideration a hierarchical classification applied on both cultured and uncultured microorganisms, 16S rRNA gene sequences were taxonomically assigned with sequence identity of 82% to orders, 86.5% to family, 94.5% to genera (Yarza et al., 2014), and 97% identity was used for species identification (Konstantinidis and Tiedje, 2005). A total of 1796 OTUs were classified into class (78.5%), order (76.8%), family (63.4%), genera (22.8%), and species (4%). A total of 3.8% of the sequences could only be assigned to the phylum Firmicutes. This result confirmed previous findings, which stated that gastrointestinal microbiota of the chicken remains largely unexplored and <200 species are isolated from chicken gastrointestinal tract (Stanley et al., 2014; Waite and Taylor, 2015). Next generation sequencing techniques have exposed the hidden diversity of microorganisms, but its taxonomic classification is difficult because of the time consuming effort to isolate and biochemically characterize individual bacteria (Yarza et al., 2014).

High variability in the microbial composition was observed between individuals (3–7 birds) within each diet and section (Table S2B). The average similarity of individuals in the studied sections ranged in the crop digesta from 29 to 82% and crop mucosa from 29 to 73%. In the ileum digesta the observed similarity of individuals was between 19 and 49% and in the ileum mucosa 25–47%. The caeca showed the lowest similarity of individuals, namely 17–38% in digesta and 30–39% in mucosa samples. The crop is dominated by *Lactobacillus* (Hagen et al., 2005; Stanley et al., 2014; Witzig et al., 2015), explaining the higher values of similarity and its simple structured microbiota when compared to other sections of the GIT. In ileum and caeca sections, the more diverse microbial communities are responsible for phytate degrading activities (Palacios et al., 2008), degrading complex organic substrates, and to the production of short chain fatty acids (SCFA; Stanley et al., 2013b; Mann et al., 2014; Choi et al., 2015). The average similarity decreased in these sections, perhaps related to the presence of a higher number of OTUs. Taking as an example diet H (with all supplements) and diet A (without any supplement), a variation in the relative abundance of predominant families was observed between the replicates in each section (Figures S1A,B). The variability between individuals has been previously reported in two studies that characterized chicken caeca (Stanley et al., 2013b; Sergeant et al., 2014) and in cattle feces (Durso et al., 2010). Furthermore, human studies found inter-individual differences in mucosa associated microbiota from colon and rectum samples (Hong et al., 2011). These studies showed that, independently of the core microbiota colonization, there is a great variation in the relative abundance of the bacterial community between individuals. A possible explanation is that shifts in microbial composition are influenced by the initially colonizing microbiota, diet, and immune system of the host (Donaldson et al., 2015).

**Figure 1:**
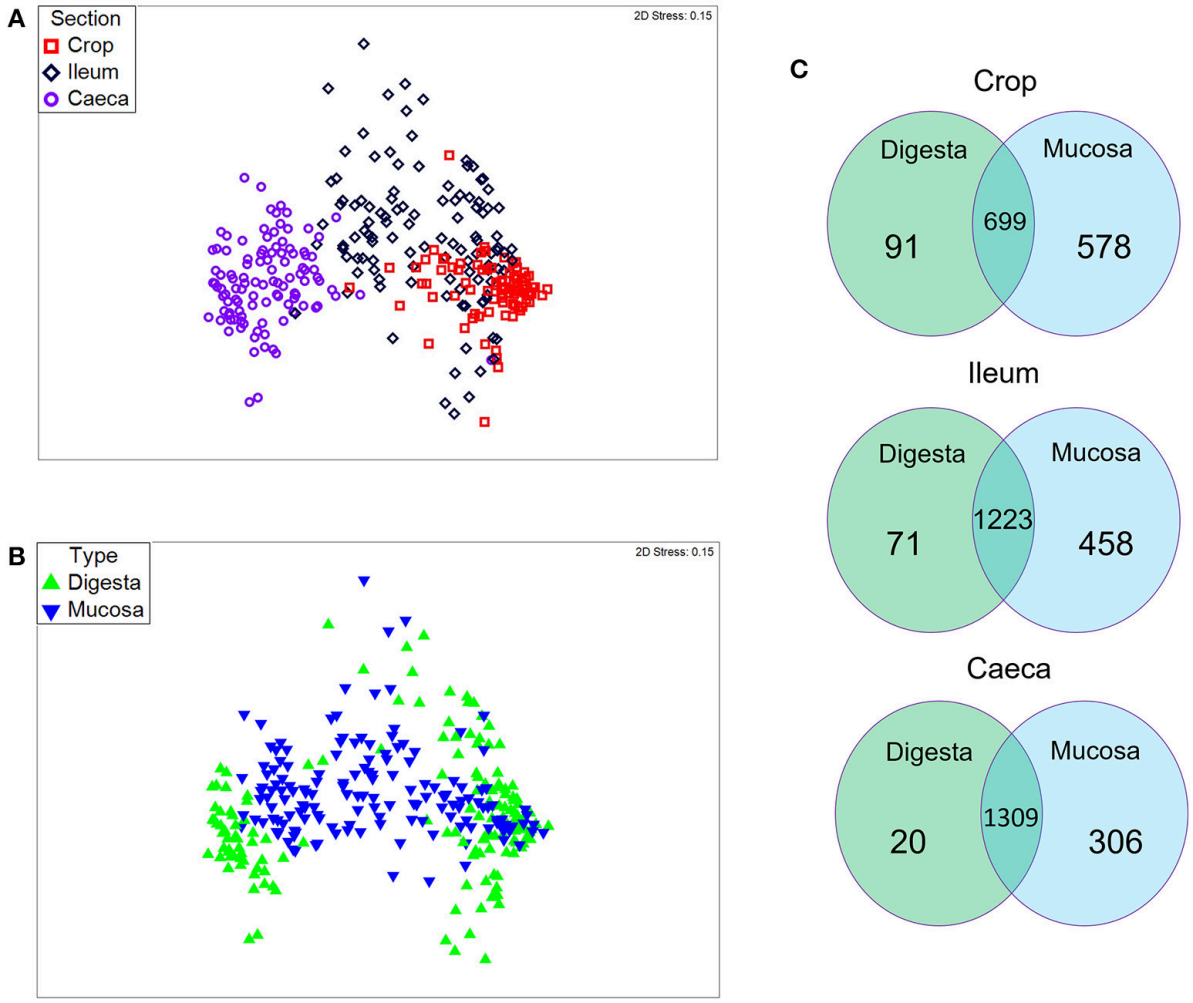
**Global bacterial community structure of 281 samples**. Sequencing data was standardized prior to the use of Bray-Curtis similarity algorithm. Non-metric multi-dimensional scaling (nMDS) plot illustrates: **(A)** crop, ileum and caeca samples, and **(B)** digesta and mucosa samples. The symbols represent a unique sample comprising all OTUs and its abundance information. **(C)** Venn diagram of the OTUs common/unique to each type of sample in the crop, ileum, and caeca. Overlapping areas show the OTUs shared between digesta and mucosa samples.

Exploring the bacterial community structure of the 281 samples, regardless of the diet, a great distinction between crop, ileum, and caeca was found to exist (*p* = 0.001; Figure 1A and Figure S2A). This confirms similar results from previous studies (Stanley et al., 2014; Witzig et al., 2015). For the first time, and in all three sections analyzed, a separation was observed between digesta and mucosa samples (*p* = 0.001; Figure 1B). Additionally, PERMANOVA results using the total number of OTUs indicated that two way interactions, diet × section and section × type of sample, were significantly different (*p* < 0.05), showing that the type of community depends on the diet and section studied and on the interactive effect of section and type of sample.

Crop samples comprised 690 OTUs shared between digesta and mucosa, a further 66 OTUs were specific to digesta and 583 OTUs to mucosa samples (Figure 1C). The diversity indices showed on average the lowest Pielou's evenness and Shannon diversity for both digesta (0.33 and 1.47, respectively) and mucosa (0.35 and 1.88, respectively), which is in accordance with previous studies (Hagen et al., 2005; Witzig et al., 2015). A similar diversity was observed in ileum digesta; however, an increase in diversity was detected in the ileum mucosa (Pielou's evenness = 0.49 and Shannon diversity = 2.9). Specific OTUs belonging to ileum digesta and mucosa samples were 64 and 490, respectively, while 1189 OTUs were observed in both (Figure 1C). The higher microbial diversity could be attributed to more suitable physicochemical conditions that allow a better establishment of complex microbiota and influence their nutrient availability (Stanley et al., 2014). Caecal digesta and mucosa samples resulted in the highest OTUs evenness (0.68 and 0.73, respectively) and diversity (4.15 and 4.6, respectively), when compared with all other sections. In the caeca digesta and mucosa 1302 OTUs were detected. A total of 24 OTUs were only detected in the digesta and 303 in the mucosa of caeca (Figure 1C). Overall, mucosa samples shared more OTUs between the three sections than digesta samples (Figure S2B). Several studies have shown that this higher diversity in the caeca is due to the low passage rate, pH, and the presence of small and soluble particles, which enhance the role of the microorganisms in assimilation of nutrients from food, in producing vitamins, and amino acids (Zhu et al., 2002; Sergeant et al., 2014), and protecting the host against pathogens (Stanley et al., 2014). Mucosa samples showed higher species diversity than digesta in all GIT sections. Most of the studies characterizing chicken microbiota have focused on digesta of the different GIT sections (Deusch et al., 2015; Waite and Taylor, 2015). The mucosa or mucous layer, which is mainly composed by mucins and glycan, help the colonization of some groups of microorganisms in the gut (Donaldson et al., 2015).

**Figure 2:**
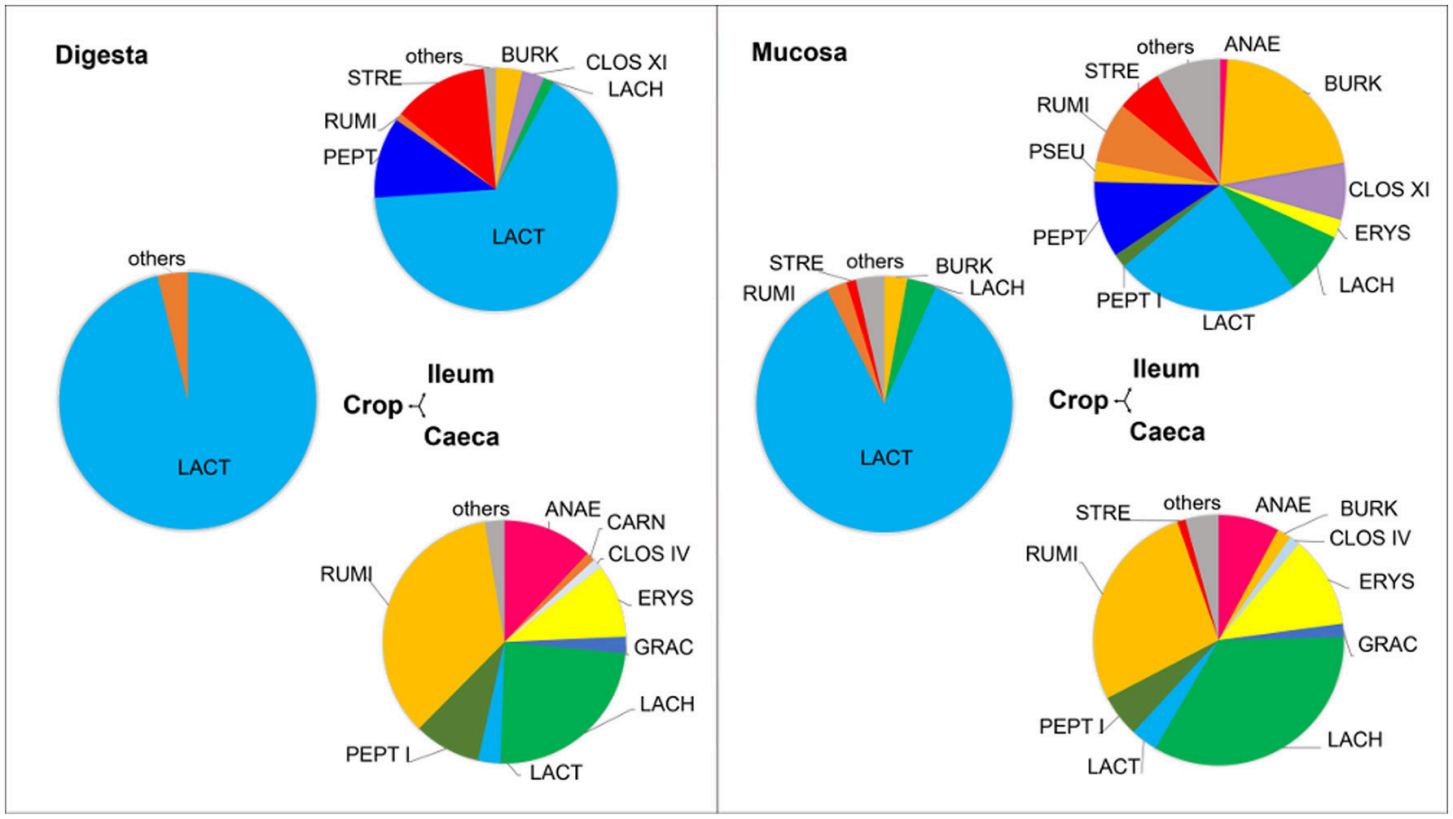
**Family distribution of digesta and mucosa samples in the crop, ileum, and caeca**. OTUs present in 281 samples were taxonomically assigned to a family and families present in abundances higher than 1% plotted. Abbreviations in the graph represent each family: ANAE, Anaeroplasmataceae; BURK, Burkholderiaceae; CARN, Carnobacteriaceae; CLOS IV, Clostridiales incertae sedis IV; CLOS XI, Clostridiales incertae sedis XI; ERYS, Erysipelotrichaceae; GRAC, Gracilibacteriaceae; LACH, Lachnospiraceae; LACT, Lactobacillus; PEPT I, Peptococcaceae I; PEPT, Peptostreptococcacaea; PSEU, Pseudomonadaceae; RUMI, Ruminococcaceae; STRE, Streptococcaceae, (Table S6).

The majority of the microorganisms colonizing the three GIT sections belonged to the phylum Firmicutes, as commonly described in previous studies that characterized the microbial communities of the chicken GIT (Stanley et al., 2013a; Deusch et al., 2015). In the crop, the most abundant family was Lactobacillaceae, which was previously reported as a dominant group in that environment (Sekelja et al., 2012; Witzig et al., 2015). Crop mucosa was additionally colonized with Lachnospiraceae, Burkholderiaceae, Ruminococcaceae, and Streptococcaceae (Figure 2). In the ileum, the dominance of Lactobacillaceae family decreased in comparison to the crop, showing 66% of abundance in digesta and 25% in the mucosa samples. The percentage of this family in the luminal content is in accordance to other broiler studies (Stanley et al., 2014; Witzig et al., 2015). However, special attention should be given to the lower abundance of Lactobacillaceae in the mucosa, which has not been reported before (Figure 2). The caeca showed higher family diversity in both digesta and mucosa samples, with similar distribution of families Ruminococcacae, Lachnospiraceae, Anaeroplasmataceae, Erysipelotrichaceae, Peptococcaceae, and Lactobacillaceae (Figure 2).

### Diet effect in the crop microbial community

**Figure 3:**
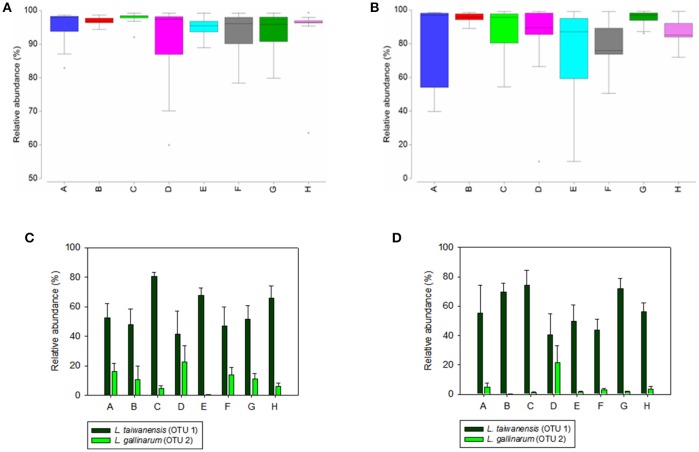
**Box-plots showing the relative abundance of the genus *Lactobacillus* in crop digesta (A)** and mucosa **(B)** across eight dietary treatments (Table 1). The box extends from the lower quartile (25%) to the higher quartile (75%). The line in the box is the median and the whiskers are the minimum and maximum values. The column charts include the relative abundances (Mean, SEM) of the two main species of *Lactobacillus, L. taiwanensis* (OTU 1), and *L. gallinarum* (OTU 2) detected in digesta **(C)** and mucosa **(D)** samples.

The composition of the microbial community of crop mucosa was significantly affected by the diets (*p* = 0.003). Such effect was not found in digesta samples, highlighting the fact that both, digesta and mucosa samples, should be studied in regard to diet effects on gut homeostasis (Figure S3A). Pair-wise comparisons showed that microbial communities of crop digesta of birds fed with diet C were significantly distinct to those derived from other diets (*p* < 0.05), with the exception of diet D (Table S4). Lower values of Shannon diversity were observed in diet C (Figure S4). This reveals a diet effect in presence of only Ca supplementation, which could be related to the lower growth and feed consumption of birds obtained with diet C (Table 2). High dietary calcium chelates part of the lipid fraction, which may reduce the energy value of the diet (Driver et al., 2005). Additionally, Ca forms insoluble complexes with phytate (Angel et al., 2002) and in the lumen interacts with inorganic phosphorus resulting in Ca-ortophosphate (Plumstead et al., 2008). Those complexes have a negative impact on the birds' performance due to the reduced solubility and availability of the P (Hamdi et al., 2015). High Ca diets have been associated with an increase of crop pH in chickens (Shafey et al., 1991) and in an higher attachment of *L. salivarius* to the GIT mucus of chickens when different *Lactobacillus* strains were studied *in vitro* (Craven and Williams, 1998), however in our study *L. taiwanensis* was the most abundant species in mucosa samples (Figures 3C,D and Table S5).

The abundance of *Lactobacillus* had the greatest fluctuation across all replicates when compared to other genera (Figures 3A,B), indicating a high variability between individuals at genus level. *Lactobacillus* was the most predominant genus in crop digesta and mucosa (Figures 3A,B and Figure S3A). Bacteria belonging to this genus efficiently colonize the squamous lining of the crop and decrease the pH due to the production of organic acids (Abbas Hilmi et al., 2007). Its presence in the gut has several advantages such as inhibition of pathogens by colonization (Abbas Hilmi et al., 2007), production of salt base hydrolase (BSH), and reduction of cholesterol concentration (Ramasamy et al., 2009). *L. taiwanensis* was the most dominant OTU in digesta and mucosa samples (OTU 1; Table S5). Birds fed with diet C showed a higher tendency to be colonized more abundantly by this OTU (74%). This result suggest that the presence of Ca favors this species. This microorganism was previously observed in the GIT of chickens fed with diets supplemented with monocalcium phosphate (Witzig et al., 2015). OTU 1 was negatively correlated with other species of *Lactobacillus* (*p* < 0.003), and a negative correlation between *L. taiwanensis* and *L. crispatus* has been previously reported in the jejunum (Witzig et al., 2015). The second most abundant OTU in crop digesta and mucosa was *L. gallinarum* (OTU 2), a homofermentative lactic acid bacterium (Hagen et al., 2005). Its abundance in crop mucosa was lower in diet B supplemented with phytase when compared to diet E, F and G (*p* < 0.05). OTU 2 was found to be negatively correlated with *L. taiwanensis* (*p* < 0.001). The *Lactobacillus acidophilus* complex, also studied in the crop (Hagen et al., 2005), consists of *L. amylovorus* (OTU 9), *L. crispatus* (OTU 11), *L. mucosae* (OTU 38), and *L. vaginalis* (OTU 25). Those OTUs revealed a propensity to be detected in lower abundance in all diets.

### Diet effects on the microbial community in the ileum

The ileum showed a higher diversity in the microbial communities when compared to the crop. Digesta samples belonging to diets C and H, that were both supplemented with Ca, were significantly different from samples derived from Ca-free diets E and F (*p* < 0.05; Table S4). It is known that higher doses of Ca in the diets can lead to an increase of the pH (Ptak et al., 2015) and low precaecal P digestibility (Adeola and Walk, 2013; Hamdi et al., 2015), which could possibly influence the presence or absence of some OTUs. An effect of P supplementation was observed in the microbial communities of the ileum mucosa. Statistical differences were obtained between diet A and F, G and H; B and F and G; diet C and F, G and H (*p* < 0.05; Table S4).

**Figure 4:**
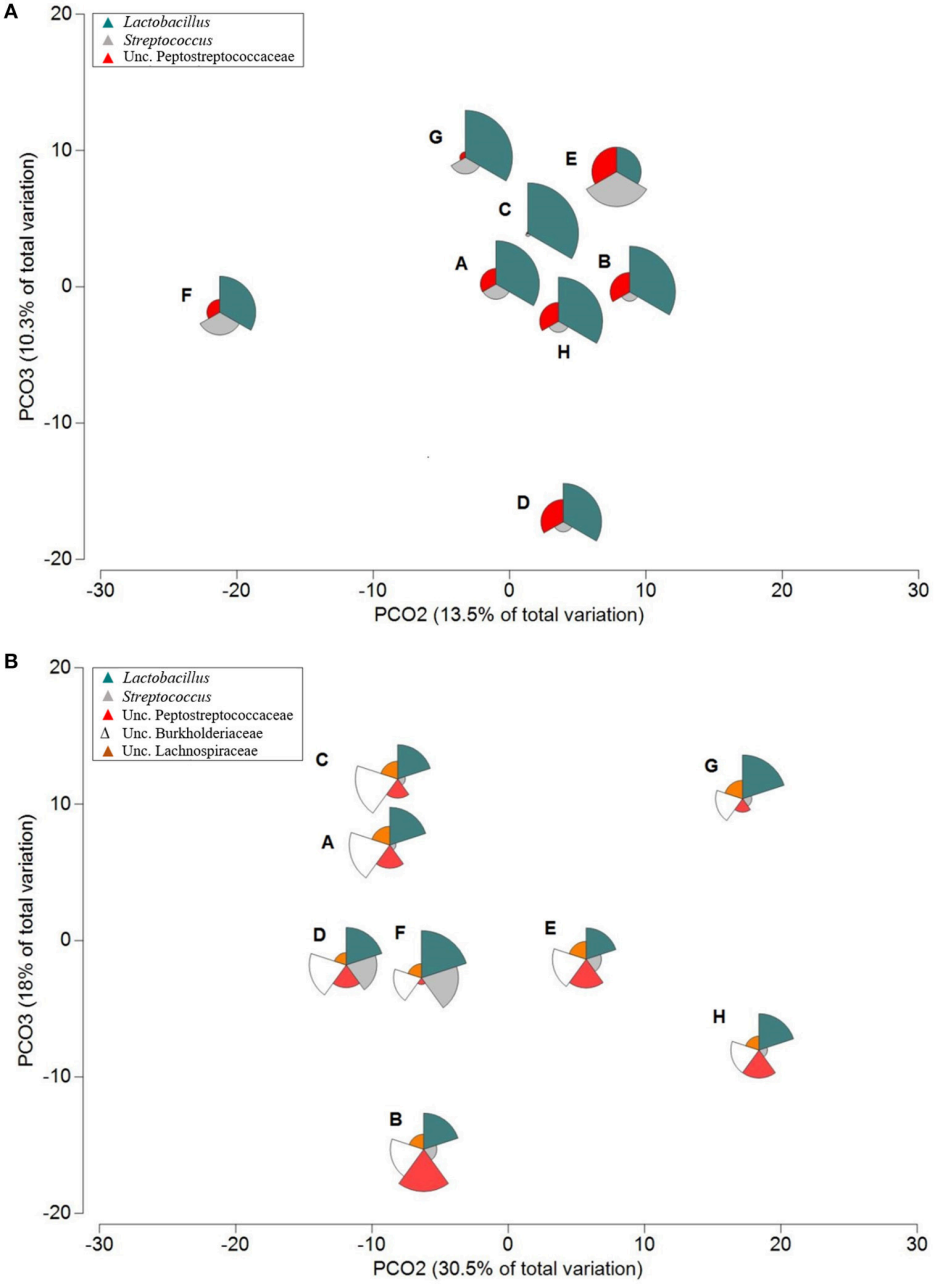
**Principal coordinate analysis (PCoA) ordination of the global bacterial community structure of ileum (A)** digesta and **(B)** mucosa samples across eight dietary treatments (A–H) (Table 1). Bubbles were superimposed to visualize the relative abundance of the most relevant genera, *Lactobacillus* and *Streptococcus* and families, Peptostreptococcaceae, Burkholderiaceae, and Lachnospiraceae (slice scale 1–100% abundance).

*Lactobacillus*, a genus widely present in crop, decreased in abundance in the ileum for most diets analyzed. The exception was for diets C and G, where it was detected at high abundances (>83%) in digesta samples. With regards to the mucosa, this genus was observed in higher abundance in diets F and G (32–37%; Figures 4A,B and Figure S3B) when compared to the other diets. Previous studies using mice and pigs have shown that diets supplemented with P and Ca, like diet G, increases *Lactobacillus* abundance (Ten Bruggencate et al., 2004; Metzler-Zebeli et al., 2010). *L. taiwanensis* (OTU 1), highly abundant in the crop, decreased its abundance in ileum digesta samples of diets supplemented with Ca (C, G, and H; 27%), while in the mucosa the highest percentage was observed on diet G (17%). The second most abundant OTU was *L. gallinarum* (OTU 2), which showed a tendency to be more abundant in diets A, C, and F (27%) for digesta and 16% in mucosa samples of diet F.

Diets E and F in digesta, and F in mucosa, both with P supplementation resulted in an increase of *Streptococcus* (44, 19, and 23%, respectively; Figures 4A,B). Lu et al. (2003) demonstrated that sequences of OTUs related to *Streptococcus* were more prevalent in the ileum digesta than in the caeca (Lu et al., 2003). In accordance with the study of Ptak et al. (2015), *Streptococcus* abundance was reduced in diets supplemented with Ca, P, and phytase (Ptak et al., 2015), represented in this study by diet H. *Streptococcus* abundance was even lower in diet C, with Ca supplementation only. OTUs assigned to uncultured Clostridium XI tended to be detected in digesta in higher abundances on diets D (18%) and E (23%) when compared to other diets, which accounted for <14%. Likewise, in the mucosa, colonization with this group mainly occurred with diet B (26%), E (14%), H (13%), and D (12%), while other diets showed abundances lower than 8%. In regards to ileum mucosa, OTUs belonging to Burkholderiaceae accounted for more than 12% of the total abundance in all dietary treatments, being detected in higher abundance in diet A and C (30%). This bacterial group showed moderate heritability in chickens, but it has not been attributed any function (Meng et al., 2014). OTUs assigned to Lachnospiraceae were commonly present in all treatments, with relative abundance ranging from 2.4 to 5.9% (Figure 4B). This family was reported to be associated with corn-based diets and is mainly composed by anaerobes and some *Clostridium* members (Munyaka et al., 2015).

*Streptococcus alactolyticus* (OTU 4) showed a tendency to be present in higher abundance in digesta samples of diets E and F with P addition (38 and 20%, respectively) and in mucosa samples of diets F and D, with phytase supplementation (22 and 13%, respectively). This lactic acid bacteria has been found in ileum samples of broilers fed with a commercial corn-soy diet (Lu et al., 2003). An uncultured Clostridium XI (OTU 7) was found with similar abundance in both digesta and mucosa samples, with the highest values observed when fed diet B (33 and 26%, respectively). Furthermore, diet B showed only 30% similarity to other diets with OTU 7 responsible for the dissimilarity. The closest relative sequence to OTU 7 was an uncultured Clostridium XI previously isolated from ileum and caeca of a conventional Ross 208 chickens grown under conditions of organic farming (Bjerrum et al., 2006). Uncultured *Ralstonia* (OTU 6), observed in the crop mucosa (<5%), showed a more prominent increase of abundance in mucosa samples for diets A and C (28 and 30%, respectively). Its abundance decreased in diets supplemented with P. A trend was detected in the increase of abundance of an OTU belonging to Clostridiaceae 1 (OTU 21) in diet F digesta (15%) and diet H mucosa (30%); which have P and phytase supplementation in common.

### Diet effect on the microbial community in the caeca

Caeca digesta and mucosa samples showed a more diverse community at genus level than observed in the other sections (Figure S3C). This fact was previously reported in chickens under standard commercial conditions (Stanley et al., 2013a; Sergeant et al., 2014; Mohd Shaufi et al., 2015) and in chickens exposed to different supplementation of monocalcium phosphate and phytase (Witzig et al., 2015). The highest OTU abundance detected in both type of samples was 14% (OTU8). Pair-wise comparison showed an effect of P in digesta samples of diet B contrasted to E, F, G, and H, but also between diet C and E (Table S4). This effect was also observed in the mucosa samples of diet B compared to F, G and H; diet D with E, F, and G; diet C with E and F, and lastly diet A and G. A high proportion of microorganisms belonging to order Clostridiales were detected in the caeca. This group is known to be an indicator of healthy chickens, due to its main role in the SCFA metabolism (Choi et al., 2015). SCFA have influence on host physiology through regulatory, immunomodulatory, and nutritional functions. They increase the growth of epithelial cells, stimulate mineral absorption and inhibit the growth and adherence of pathogenic microorganisms by decreasing the pH (Walugembe et al., 2015).

**Figure 5:**
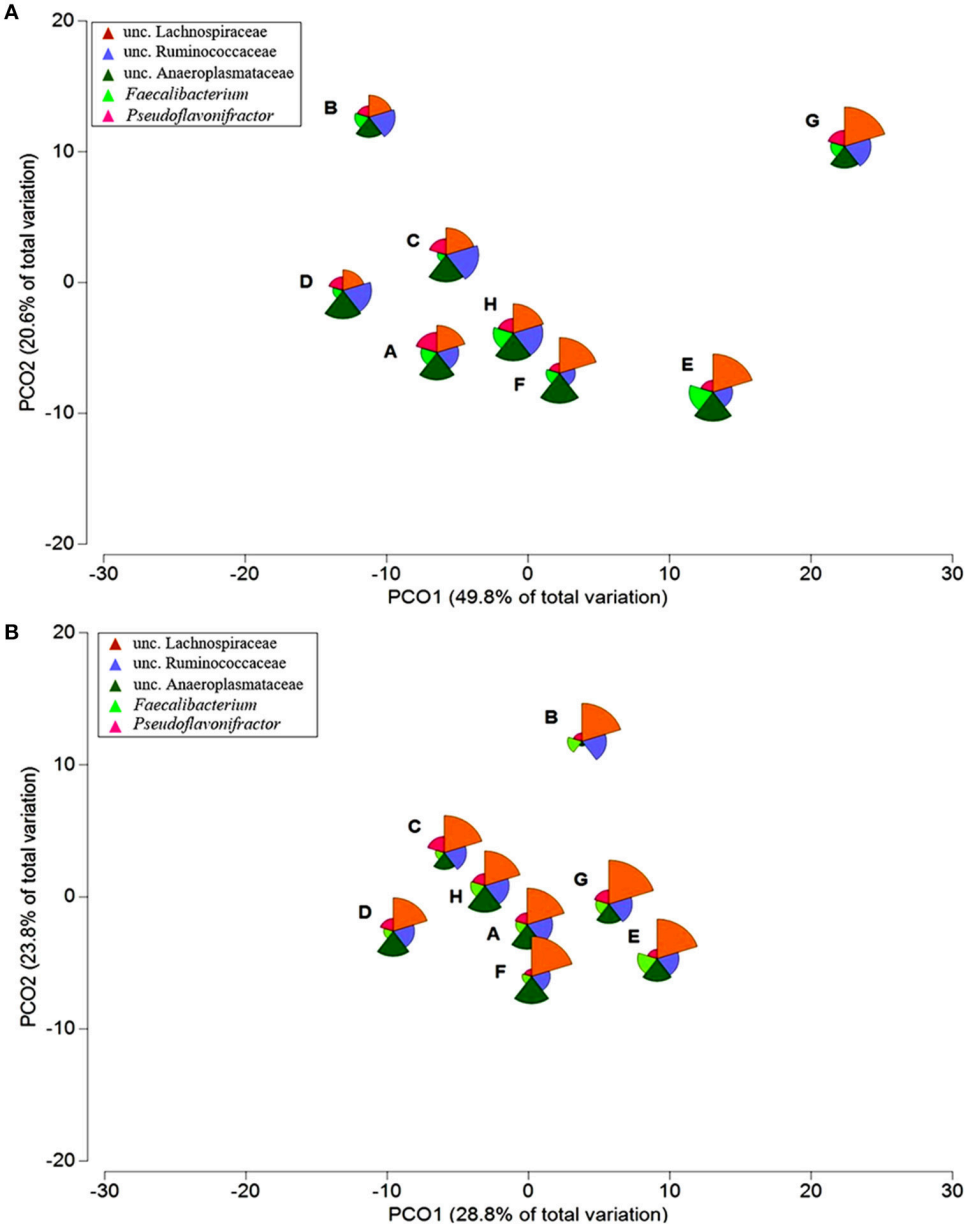
**Principal coordinate analysis (PCoA) ordination of the global bacterial community structure of caeca (A)** digesta and **(B)** mucosa samples across eight dietary treatments (A–H) (Table 1). Bubbles were superimposed to visualize the relative abundance of the most relevant genera, *Faecalibacterium* and *Pseudoflavonifractor* and families, Lachnospiraceae, Ruminococcaceae, and Anaeroplasmataceae (slice scale 1–30% abundance).

OTUs belonging to Lachnospiraceae are known to degrade complex polysaccharides to SCFA (Biddle et al., 2013). They were more abundant in digesta samples of diets supplemented with P (12–22%), while in the mucosa showed a similar distribution within all diets (17–28%; Figure 5 and Figure S3C). Ruminococcaceae is a common family reported in the chicken caeca (Bjerrum et al., 2006; Mohd Shaufi et al., 2015) and it was detected in both digesta (4–8%) and mucosa (3–13%) samples. Both families have been associated with the maintenance of gut health and have the enzymatic capability to degrade cellulose and hemicellulose (Biddle et al., 2013). Erysipelotrichaceae showed an abundance of 2% in the digesta samples of diets supplemented with P, however in the mucosa a higher abundance was detected (3–8%). In the caeca, protein sequences related to butyryl-CoA production enzymes have been previously detected on this family (Eeckhaut et al., 2011; De Maesschalck et al., 2014). One group of OTUs, closely related to the family Anaeroplasmataceae, were observed in all diets (Figure 5). This family has been reported in the chicken gastrointestinal microbiome (Oakley et al., 2014), but the exact role in chicken GIT remains unknown. A species belonging to Anaeroplasmataceae was previously described in rumen samples and related to bacteriolytic and non-bacteriolytic activities (Robinson et al., 1975). This can explain the negative correlation of OTU 8 (uncultured *Anaeroplasma*) with other OTUs in digesta and mucosa samples such as OTU 394 (uncultured Lachnospiraceae), OTU 116 (uncultured Clostridium XIVa), OTU 390 (uncultured Ruminococcaceae), and OTU 93 (uncultured *Faecalibacterium*; *p* < 0.05).

The OTUs in digesta samples related to *Lactobacillus* were more abundant when fed diet G (14.8%), with P and Ca additions (Figure S3C), with *L. gallinarum* (OTU 2; 12%) and *L. taiwanensis* (OTU 1; 2%) as the main colonizers. However, in the other diets, these OTUs were present in abundances lower than 2%. This is in accordance with a recent metagenomic study on the chicken caeca that showed *Lactobacillus* in low abundances (<4%; Mohd Shaufi et al., 2015). Diet E, with P supplementation, showed a group of OTUs closely related to *Faecalibacterium* in both type of samples. This genus is one of the most prominent butyrate producers, providing energy to the colonic mucosa and known to regulate gene expression, inflammation, differentiation, and apoptosis in host cells (Luo et al., 2013). *Pseudoflavonifractor*, detected in digesta and mucosa, is a common caeca colonizer that has a protein from class IV alcohol dehydrogenase that influences the final butyrate production pathway (Polansky et al., 2015). *Erysipelotrichaceae incertae sedis* previously reported in chicken caeca (Stanley et al., 2012) was detected more consistently throughout the diets in digesta samples and the same applied to *Streptococcus* in the mucosa.

Supplementation of Ca in diet C enhanced the presence of OTU 45 (5%) in caeca digesta. This OTU is related to an uncultured *Subdoligranulum* sp. that was previously found in the caeca of turkeys (Scupham, 2007) and is capable of producing butyric acid. OTU 37, an uncultured Ruminococcaceae, was detected in lower abundance (3%) in diets without P supplementation (A to D) or P with phytase supplementation (F) and has been previously detected in the intestinal microbiota of preadolescent turkeys (Scupham, 2007). In the caeca mucosa samples, an OTU with high similarity to an uncultured Bacillales (OTU 23) was found. This OTU was present in higher abundance on diet B (6.7%), with phytase supplementation, when compared to diets A, E, and F (4.5, 3.1, and 1.8%, respectively; *p* < 0.05). Particularly, this OTU was negatively correlated with OTU 31, related to an uncultured Lachnospiraceae, and OTU 91 related to an uncultured Ruminococcaceae (*p* < 0.05). Furthermore, OTU 4 identified as *Streptococcus alactolyticus* and highly abundant in some ileum samples, decreased its abundance in the caeca being 1.5% the highest value observed. This result contradicts a previous study on broilers fed diets including peas and organic acids where *S. alactolyticus* was a dominant species (Czerwiñski et al., 2010).

In mucosa samples, the abundance of OTUs belonging to the Clostridium XIVa and XIVb was higher than in digesta. The first family comprises some microorganisms that are butyrate producers while the second includes propionate producers and therefore may be linked to beneficial effects in the GIT (De Maesschalck et al., 2015). An uncultured Clostridium XIVb (OTU 56) previously found in caeca of preadolescent turkeys (Scupham, 2007), was present in birds fed diets B, C, D, E, and F (2.5–3%). OTU 81, similar to uncultured Clostridium XIVb, was positively correlated with OTU 56 (*p* < 0.05) and was previously reported to be present in the human ileum (Li et al., 2012). OTU 87, an uncultured Clostridium XIVa found in human feces (Turnbaugh et al., 2009), was more abundant on diet A and F, without calcium supplementation when compared to diet D, supplemented with Ca (*p* < 0.05).

It is known that non-ruminant animals are not efficient in utilizing phytate-P. In this study we have found, in the ileum and caeca, OTUs related to the genus *Clostridium*, which have been previously isolated and associated to the production of cysteine phytase (Gruninger et al., 2009). *Megasphaera elsdenii* (OTU 111) and *Mitsuokella* spp. (OTU 1501), common members of the rumen microbiota that have the ability to produce phytases (Yanke et al., 1998), were also detected in the ileum and caeca samples from birds on diets supplemented with Ca, P, or P with phytase.

## Conclusions

Diet supplementation with P, Ca, or phytase has an effect on the microbial community that colonizes the GIT. However, a consistent effect of diet on the microbiota harbored in the different sections of the GIT was not observed. This was likely due to the high variability between individuals. Lower microbial diversity was associated with lower growth performance in animals fed with a diet only supplemented with Ca. Diets supplemented with P influenced the caeca microbiota and positively affected the growth of the broilers. For a better understanding of dietary effects on broiler performance, gut function and balance, and the microbial community, digesta and mucosa samples should be studied in separate as both showed different microbial communities.

## Authors contributions

Conceived and designed the experiment: AC, VS, MR. Performed the experiments: DB. Bioinformatics analysis: MV. OTUs annotation: DB. Data analysis: DB, AC. Performance data analysis: VS. Wrote the paper: DB, AC. Article revision and final approval: MV, VS, MR, AC.

## Funding

This project has been funded in part by the Ministerium für Wissenschaft, Forschung und Kunst Baden-Württemberg, Stuttgart, Germany.

### Conflict of interest statement

The authors declare that the research was conducted in the absence of any commercial or financial relationships that could be construed as a potential conflict of interest.

## Supplementary material

Supplementary Table 1 **Dietary composition of the commercial starter diet fed until day 14 and basal diet for the corresponding treatments with P, Ca and phytase supplementation fed from days 15 to 26**.

Supplementary Table 2 **Description of the three GIT sections in regards to (A)** number of replicates per diet and type of sample and **(B)** average similarity of the replicates.

Supplementary Table 3 **OTUs abundances across the eight dietary treatments and the GIT sections**.

Supplementary Table 4 **Statistical differences between the sections and the type of samples based on PERMANOVA results**. Pairwise comparison results of the diets that showed a significant difference.

Supplementary Table 5 **Taxonomic assignment of the most relevant OTUs present in the chicken gastrointestinal tract**. The assignment was performed in the Seqmatch function of the RDP database for type and non-type strain.

Supplementary Table 6 **Percentages of the families present in crop, ileum and caeca for digesta and mucosa**.

Supplementary Figure 1 **Shade plot showing the relative abundance of each family present on each replicate of crop, ileum and caeca mucosa samples of (A)** diet H (Ca, P, and phytase supplementation) and **(B)** diet A (no supplementation). The intensity of the color increases to black if the family was detected in higher abundance, while white indicates family absence.

Supplementary Figure 2 **(A)** Non-metric multi-dimensional scaling (nMDS) plot to illustrates the three GIT sections crop, ileum and caeca samples, splitted by the type of sample digesta and mucosa. The symbols represent a unique sample comprising all OTUs and its abundance information. **(B)** Venn diagrams of the OTUs common/unique to the type of samples digesta and mucosa in the three GIT sections: crop, ileum and caeca. Overlapping areas show the OTUs commonly shared.

Supplementary Figure 3 **Bar plots showing the relative abundance of the genus detected in digesta and mucosa samples in the eight dietary treatments (A)** crop, **(B)** ileum, and **(C)** caeca.

Supplementary Figure 4 **Diversity observed across the three GIT sections studied: crop, ileum, and caeca and the two type of samples: digesta and mucosa, for the eight dietary treatments**. Values are calculated based on the Shannon diversity index.

## Abbreviations

P: Phosphorus
Ca: calcium
GIT: gastrointestinal tract
kg: kilograms
FTU: phytase unit
CO_2_: carbon dioxide
N_2_: nitrogen
O_2_: oxygen
cm: centimeters
°C: Celsius degrees
BW: body weight
FC: feed consumption
PCR: polymerase chain reaction
bp: base pairs
OTU: operational taxonomic units
RDP: ribosomal database project
ENA: European Nucleotide Archive
nMDS: non-metric multidimensional scaling plots
PcoA: principal coordinate analysis
SIMPER: similarity percentages analysis
PERMANOVA: permutational manova H′, Shannon-weaver index of diversity
SCFA: short chain fatty acids
ANAE: Anaeroplasmataceae
BURK: Burkholderiaceae
CARN: Carnobacteriaceae
CLOS IV: *Clostridiales incertae sedis* IV
CLOS XI: *Clostridiales incertae sedis* XI
ERYS: Erysipelotrichaceae
GRAC: Gracilibacteriaceae
LACH: Lachnospiraceae
LACT: *Lactobacillus*
PEPT I: Peptococcaceae I
PEPT: Peptostreptococcaceae
PSEU: Pseudomonadaceae
RUMI: Ruminococcaceae
STRE: Streptococcaceae.
