# Supplementary material for: Insights into Broilers' Gut Microbiota Fed with Phosphorus, Calcium, and Phytase Supplemented Diets
Source: Front Microbiol. 2016 Dec 19;7:2033. doi: 10.3389/fmicb.2016.02033 (PMC5165256; doi:10.3389/fmicb.2016.02033)
Supplement: Supplementary Table 1 — Dietary composition of the commercial starter diet fed until day 14 and basal diet for the corresponding treatments with P, Ca and phytase supplementation fed from days 15 to 26. [file Table1.DOCX]

**Table S1**. Dietary composition of the commercial starter diet fed until day 14 and basal diet for the corresponding treatments with P, Ca and phytase supplementation fed from days 15 to 26.

| **Starter Diet Composition** | | | | |
| --- | --- | --- | --- | --- |
| **Composition** | |  | **Additives** | |
| Energy (ME) | 12.2 MJ |  | Vitamin A | 15,000 I.E. |
| Crude protein | 22% |  | Vitamin D_3_ | 5,000 I.E. |
| Methionine | 0.62% |  | Vitamin E | 100 mg |
| Crude fat | 5.50% |  | Zinc | 60 mg |
| Crude fiber | 3% |  | Manganese | 60 mg |
| Ash | 6.10% |  | Copper | 6 mg |
| Calcium | 0.95% |  | Iron | 30 mg |
| Phosphorus | 0.65% |  | Iodine | 0.36 mg |
| Sodium | 0.16% |  | Selenium | 0.3 mg |
|  |  |  | Coccidiostat | + |
|  |  |  |  |  |
| **Dietary treatments** | | | | |
| Ingredient, g/kg as fed | P- Ca- | P- Ca+ | P+ Ca- | P+ Ca+ |
| Maize | 541 | 541 | 541 | 541 |
| Extracted soybean meal (48%) | 400 | 400 | 400 | 400 |
| Soybean oil | 15 | 15 | 15 | 15 |
| D,L-Methionine | 2 | 2 | 2 | 2 |
| Monosodium phosphate | - | - | 10.5 | 10.5 |
| Sand | 18 | 8.5 | 9.5 | - |
| Limestone (fine) | 10 | 19.5 | 10 | 19.5 |
| Sodium chloride | 1 | 1 | 1 | 1 |
| Cholin chloride | 2 | 2 | 2 | 2 |
| Sodium bicarbonate | 3 | 3 | 1 | 1 |
| Vitamine mix | 2 | 2 | 2 | 2 |
| Mineral mix | 1 | 1 | 1 | 1 |
| TiO_2_ | 5 | 5 | 5 | 5 |
| Calculated composition, g/kg DM | | | | |
| Crude protein | 251 | 251 | 251 | 251 |
| Lysine | 14 | 14 | 14 | 14 |
| Methionine | 4.7 | 4.7 | 4.7 | 4.7 |
| Methionine+Cysteine | 10.3 | 10.3 | 10.3 | 10.3 |
| ME (MJ/kg) | 13.5 | 13.5 | 13.5 | 13.5 |
| Total-P | 4.1 | 4.1 | 6.9 | 6.9 |
| Ca | 6.2 | 10.3 | 6.2 | 10.3 |
