# Supplementary material for: Insights into Broilers' Gut Microbiota Fed with Phosphorus, Calcium, and Phytase Supplemented Diets
Source: Front Microbiol. 2016 Dec 19;7:2033. doi: 10.3389/fmicb.2016.02033 (PMC5165256; doi:10.3389/fmicb.2016.02033)
Supplement: Supplementary Table 2 — Description of the three GIT sections in regards to (A) number of replicates per diet and type of sample and (B) average similarity of the replicates. [file Table2.DOCX]

**Table S2.** Description of the three GIT sections in regards to (a) number of replicates per diet and type of sample and (b) average similarity of the replicates.

1. Number of replicates

| Diet | Crop | | Ileum | | Caeca | |
| --- | --- | --- | --- | --- | --- | --- |
|  | Digesta | Mucosa | Digesta | Mucosa | Digesta | Mucosa |
| A | 5 | 3 | 6 | 7 | 3 | 7 |
| B | 5 | 4 | 4 | 6 | 7 | 4 |
| C | 7 | 4 | 5 | 7 | 7 | 7 |
| D | 5 | 6 | 6 | 6 | 4 | 7 |
| E | 3 | 4 | 6 | 7 | 7 | 7 |
| F | 4 | 6 | 7 | 7 | 6 | 7 |
| G | 6 | 6 | 7 | 7 | 7 | 7 |
| H | 6 | 5 | 7 | 7 | 7 | 6 |

1. Average similarity

| Diet | Crop | | Ileum | | Caeca | |
| --- | --- | --- | --- | --- | --- | --- |
|  | Digesta | Mucosa | Digesta | Mucosa | Digesta | Mucosa |
| A | 58 % | 34 % | 33 % | 47 % | 24 % | 35 % |
| B | 51 % | 73 % | 24 % | 38 % | 29 % | 32 % |
| C | 82 % | 69 % | 40 % | 41 % | 30 % | 33 % |
| D | 29 % | 29 % | 19 % | 25 % | 28 % | 39 % |
| E | 72 % | 56 % | 36 % | 36 % | 26 % | 30 % |
| F | 43 % | 56 % | 23 % | 26 % | 28 % | 33 % |
| G | 54 % | 73 % | 42 % | 29 % | 17 % | 31 % |
| H | 69 % | 69 % | 49 % | 36 % | 39 % | 35 % |
