# Supplementary material for: Insights into Broilers' Gut Microbiota Fed with Phosphorus, Calcium, and Phytase Supplemented Diets
Source: Front Microbiol. 2016 Dec 19;7:2033. doi: 10.3389/fmicb.2016.02033 (PMC5165256; doi:10.3389/fmicb.2016.02033)
Supplement: Supplementary Table 4 — Statistical differences between the sections and the type of samples based on PERMANOVA results. Pairwise comparison results of the diets that showed a significant difference. [file Table4.DOCX]

**Table S4.** Statistical differences between the sections and the type of samples based on PERMANOVA results. Pairwise comparison results of the diets that showed a significant difference.

PERMANOVA results for the type of sample

|  | Groups | t | P(perm) | Unique perms |
| --- | --- | --- | --- | --- |
| Crop | Digesta, Mucosa | 4.358 | 0.001 | 999 |
| Ileum | Digesta, Mucosa | 4.358 | 0.001 | 999 |
| Caeca | Digesta, Mucosa | 3.182 | 0.001 | 998 |

Pairwise test

| Section | Type | Groups | t | P(perm) | Unique perms |
| --- | --- | --- | --- | --- | --- |
| Crop | Digesta | C, E | 1.563 | 0.042 | 120 |
|  |  | C, H | 1.561 | 0.041 | 753 |
|  |  | C, A | 2.099 | 0.011 | 549 |
|  |  | C, F | 1.937 | 0.04 | 313 |
|  |  | C, G | 2.052 | 0.014 | 761 |
|  |  | C, B | 1.92 | 0.007 | 566 |
| Ileum | Digesta | C, E | 2.109 | 0.009 | 401 |
|  |  | E, H | 2.02 | 0.021 | 769 |
|  |  | E, G | 1.997 | 0.016 | 768 |
|  |  | E, A | 1.663 | 0.049 | 421 |
|  |  | H, F | 0.3722 | 0.047 | 752 |
| Ileum | Mucosa | A, F | 1.538 | 0.033 | 785 |
|  |  | A, G | 1.546 | 0.024 | 754 |
|  |  | C, H | 1.537 | 0.009 | 766 |
|  |  | C, F | 1.521 | 0.035 | 741 |
|  |  | C, G | 1.435 | 0.034 | 760 |
|  |  | E, F | 1.465 | 0.045 | 737 |
|  |  | F, B | 1.555 | 0.035 | 765 |
|  |  | G, B | 1.494 | 0.023 | 780 |
|  |  | H, A | 1.627 | 0.009 | 753 |

| Section | Type | Groups | t | P(perm) | Unique perms |
| --- | --- | --- | --- | --- | --- |
| Caeca | Digesta | C, E | 1.205 | 0.03 | 763 |
|  |  | E, B | 1.204 | 0.042 | 763 |
|  |  | F, B | 1.296 | 0.02 | 750 |
|  |  | G, B | 1.242 | 0.008 | 762 |
|  |  | H, B | 1.3 | 0.016 | 748 |
|  |  | H, F | 1.286 | 0.044 | 743 |
| Caeca | Mucosa | A, G | 1.191 | 0.027 | 751 |
|  |  | A, B | 1.187 | 0.046 | 319 |
|  |  | C, E | 1.235 | 0.022 | 765 |
|  |  | C, F | 1.306 | 0.014 | 756 |
|  |  | D, B | 1.275 | 0.022 | 317 |
|  |  | D, E | 1.272 | 0.018 | 737 |
|  |  | D, F | 1.265 | 0.034 | 766 |
|  |  | D, G | 1.373 | 0.003 | 768 |
|  |  | F, G | 1.291 | 0.02 | 751 |
|  |  | F, B | 1.378 | 0.026 | 312 |
|  |  | G, B | 1.251 | 0.011 | 314 |
|  |  | H, B | 1.283 | 0.009 | 209 |
|  |  | H, F | 1.241 | 0.038 | 763 |
