# Supplementary material for: Insights into Broilers' Gut Microbiota Fed with Phosphorus, Calcium, and Phytase Supplemented Diets
Source: Front Microbiol. 2016 Dec 19;7:2033. doi: 10.3389/fmicb.2016.02033 (PMC5165256; doi:10.3389/fmicb.2016.02033)
Supplement: Supplementary Table 5 — Taxonomic assignment of the most relevant OTUs present in the chicken gastrointestinal tract. The assignment was performed in the Seqmatch function of the RDP database for type and non-type strain. [file Table5.DOCX]

**Table S5**. Taxonomic assignment of the OTUs described in the text and present in the chicken gastrointestinal tract. The assignment was performed in the Seqmatch function of the RDP database for type and non-type strain.

| OTU | Closest reference strain (GenBank accesion N°) | Similarity |
| --- | --- | --- |
| 1 | *Lactobacillus taiwanensis* (EU487512) | 98.2% |
| 2 | *Lactobacillus gallinarum* (EF412984) | 100% |
| 4 | *Streptococcus alactolyticus* (AF201899) | 100% |
| 6 | Uncultured *Ralstonia (JQ794615)* | 100% |
| 7 | Uncultured *Clostridium* XI (DQ057389) | 100% |
| 8 | Uncultured *Anaeroplasma* (GQ175516) | 100% |
| 9 | *Lactobacillus amylovorus* (AY700063) | 100% |
| 11 | *Lactobacillus crispatus* (AF257097) | 100% |
| 21 | Uncultured *Clostridiaceae* 1 (DQ342328) | 100% |
| 23 | Uncultured *Bacillales (*AB265205) | 100% |
| 25 | *Lactobacillus vaginalis* (HQ293058) | 98.5% |
| 31 | Uncultured *Lachnospiraceae* (EF025277) | 100% |
| 32 | *Lactobacillus reuteri* (EU722746) | 100% |
| 34 | *Lactobacillus vaginalis* (AB911497) | 97.9% |
| 37 | Uncultured *Ruminococcaceae* (DQ455830) | 100% |
| 38 | *Lactobacillus mucosae* (EU728797) | 98.5% |
| 45 | Uncultured *Subdoligranulum* sp. (FJ440072) | 100% |
| 46 | Uncultured *Clostridium* XVIII (AY984680) | 100% |
| 56 | Uncultured *Clostridium* XIVb (DQ455825) | 97.7% |
| 81 | Uncultured *Clostridium* XIVb (HQ821269) | 100% |
| 87 | Uncultured Clostridium XIVa (FJ366976) | 97.1% |
| 91 | Uncultured *Ruminococcacae* (DQ793581) | 100% |
| 93 | Uncultured *Faecalibacterium* (DQ4558359 | 95.4% |
| 111 | *Megasphaera elsdenii* (AY196919) | 100% |
| 116 | Uncultured *Clostridium* XIVa (DQ800343) | 100% |
| 390 | Uncultured *Ruminococcaceae* (FJ365262) | 87.5% |
| 394 | Uncultured *Lachnospiraceae* (DQ673545) | 91.5% |
| 508 | *Lactobacillus oris (*AB425929) | 100% |
| 1501 | *Mitsuokella jalaludinii* (AF479674) | 94% |
