# Supplementary material for: Insights into Broilers' Gut Microbiota Fed with Phosphorus, Calcium, and Phytase Supplemented Diets
Source: Front Microbiol. 2016 Dec 19;7:2033. doi: 10.3389/fmicb.2016.02033 (PMC5165256; doi:10.3389/fmicb.2016.02033)
Supplement: Supplementary Table 6 — Percentages of the families present in crop, ileum and caeca for digesta and mucosa. [file Table6.DOCX]

**Table S6**. Percentages of the families present in crop, ileum and caeca for digesta and mucosa.

|  | **Crop** | | **Ileum** | | **Caeca** | |
| --- | --- | --- | --- | --- | --- | --- |
|  | Digesta | Mucosa | Digesta | Mucosa | Digesta | Mucosa |
| *Lactobacillaceae* (LACT) | 96.2 | 86 | 66 | 25.2 | 3 | 3.4 |
| *Ruminococcaceae* (RUMI) | - | 2.6 | 1 | 8.3 | 35 | 27 |
| *Lachnospiraceae* (LACH) | - | 3.7 | 1.4 | 8.8 | 24 | 34.2 |
| *Burkholderiaceae* (BURK) | - | 2.9 | 3.5 | 22.5 | - | 1.6 |
| *Streptococcaceae* (STRE) | - | 1.2 | 12.7 | 6.2 | - | 1.1 |
| *Peptostreptococcaceae* (PEPT) | - | - | 10.8 | 10.4 | - | - |
| *Clostridiales* Incertae Sedis XI (CLOS XI) | - | - | 3 | 7.8 | 1.3 | 1.3 |
| *Pseudomonadaceae* (PSEU) | - | - | - | 2.8 | - | - |
| *Erysipelotrichaceae* (ERYS) | - | - | - | 2.5 | 9.8 | 11.7 |
| *Peptococcaceae 1* (PEPT I) | - | - | - | 1.9 | 8.9 | 5.6 |
| *Anaeroplasmataceae* (ANAE) | - | - | - | 1 | 12 | 8.2 |
| *Gracillibacteriaceae* (GRAC) | - | - | - | - | 2.2 | 1.9 |
| *Carnobacteriaceae* (CARN) | - | - | - | - | 1.2 | - |
| Others | 3.8 | 3.6 | 1.6 | 2.6 | 2.6 | 4 |
