# Supplementary figures and images for: Insights into Broilers' Gut Microbiota Fed with Phosphorus, Calcium, and Phytase Supplemented Diets
Source: Front Microbiol. 2016 Dec 19;7:2033. doi: 10.3389/fmicb.2016.02033 (PMC5165256; doi:10.3389/fmicb.2016.02033)

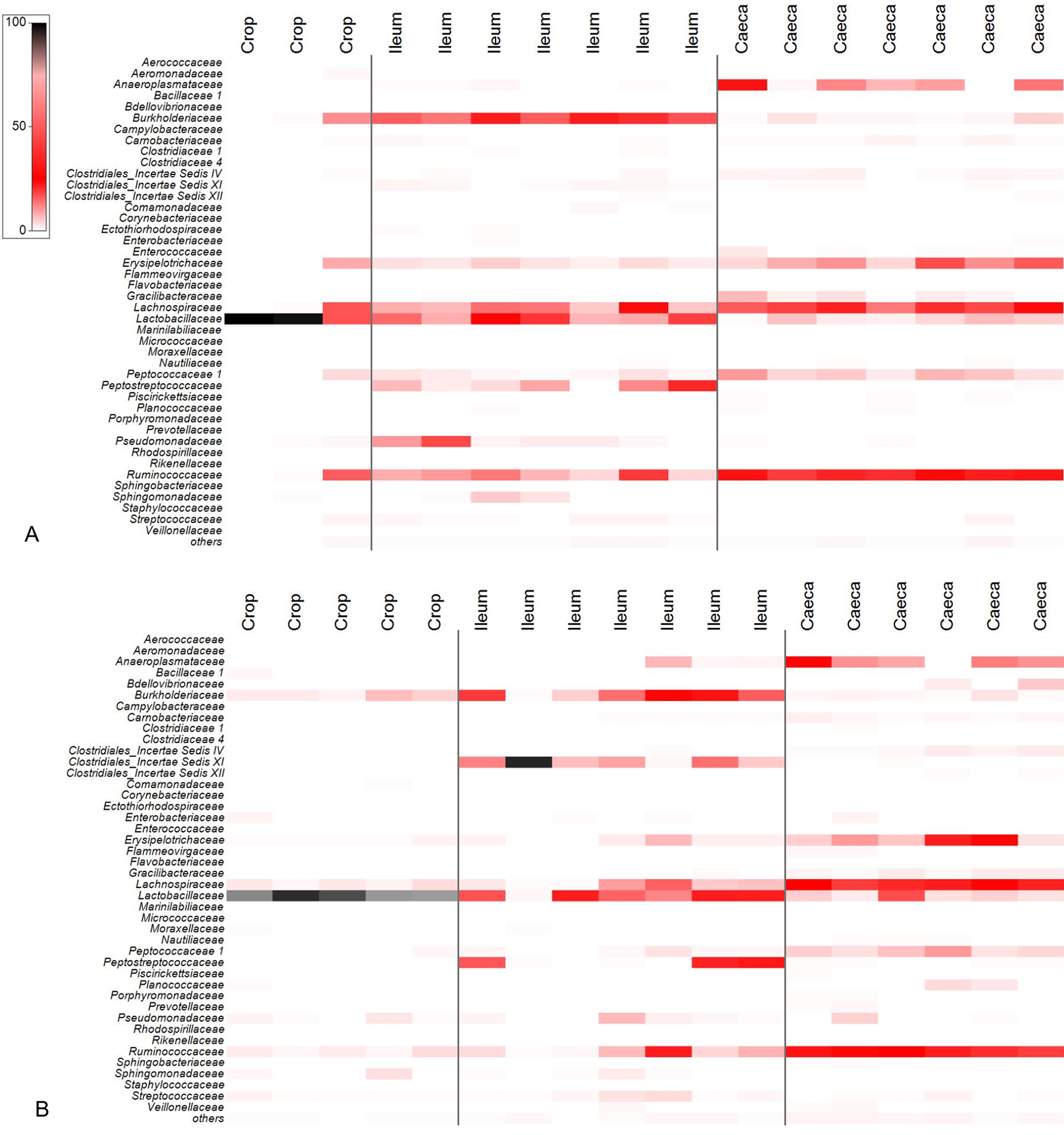

Supplement: Supplementary Figure 1 — Shade plot showing the relative abundance of each family present on each replicate of crop, ileum and caeca mucosa samples of (A) diet H (Ca, P, and phytase supplementation) and (B) diet A (no supplementation). The intensity of the color increases to black if the family was detected in higher abundance, while white indicates family absence. [file Image1.TIF]

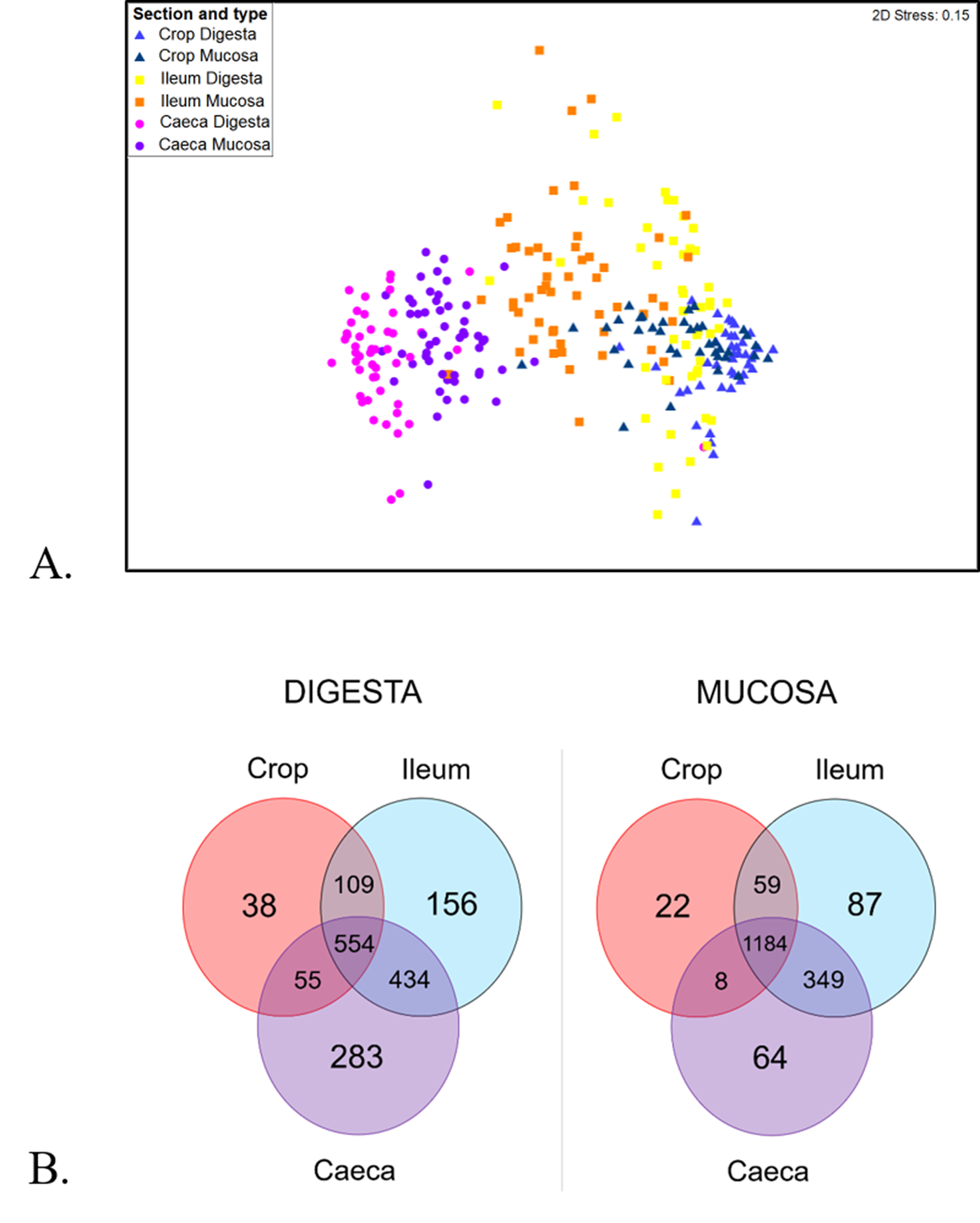

Supplement: Supplementary Figure 2 — (A) Non-metric multi-dimensional scaling (nMDS) plot to illustrates the three GIT sections crop, ileum and caeca samples, splitted by the type of sample digesta and mucosa. The symbols represent a unique sample comprising all OTUs and its abundance information. (B) Venn diagrams of the OTUs common/unique to the type of samples digesta and mucosa in the three GIT sections: crop, ileum and caeca. Overlapping areas show the OTUs commonly shared. [file Image2.TIF]

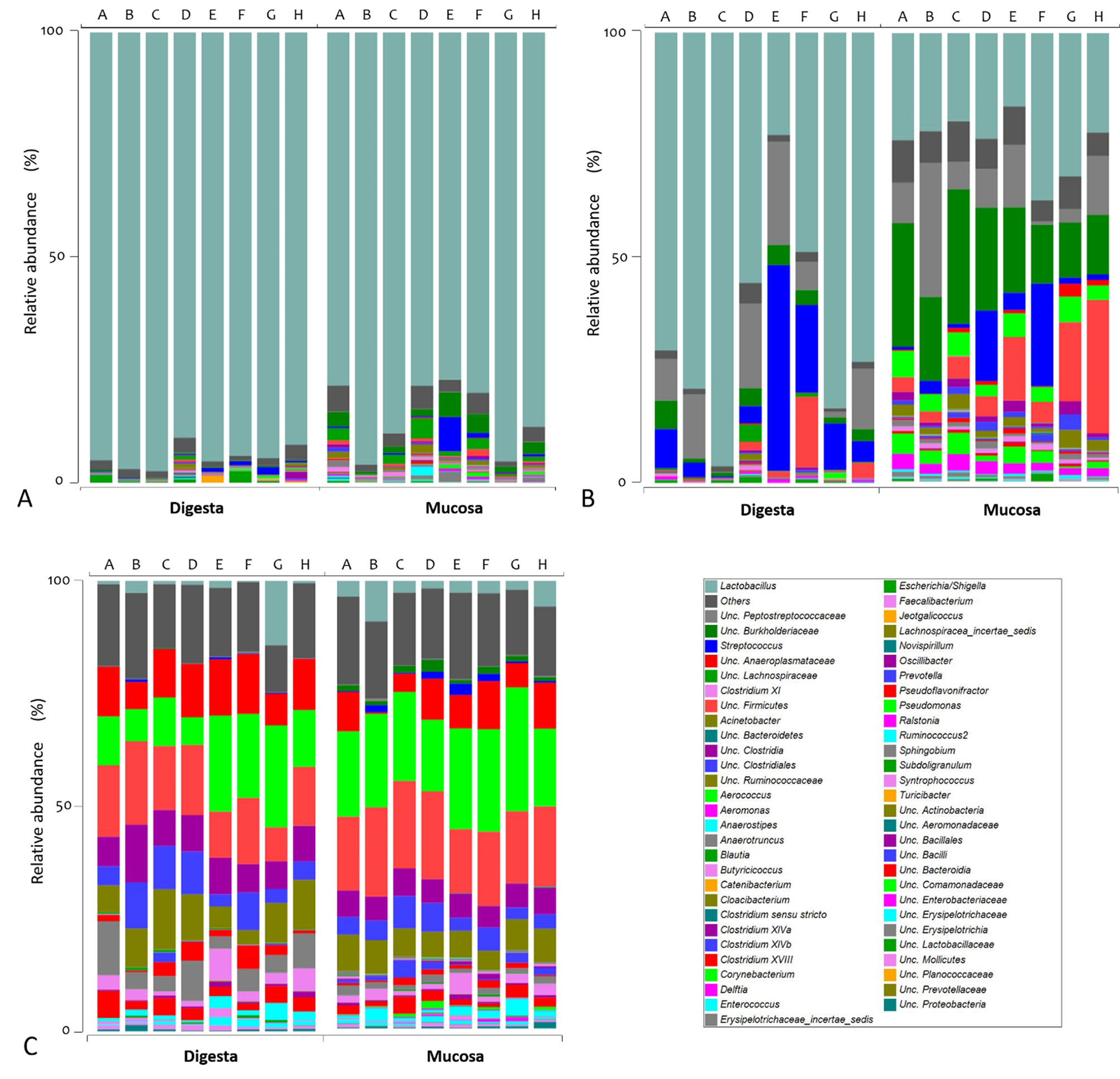

Supplement: Supplementary Figure 3 — Bar plots showing the relative abundance of the genus detected in digesta and mucosa samples in the eight dietary treatments (A) crop, (B) ileum, and (C) caeca. [file Image3.TIF]

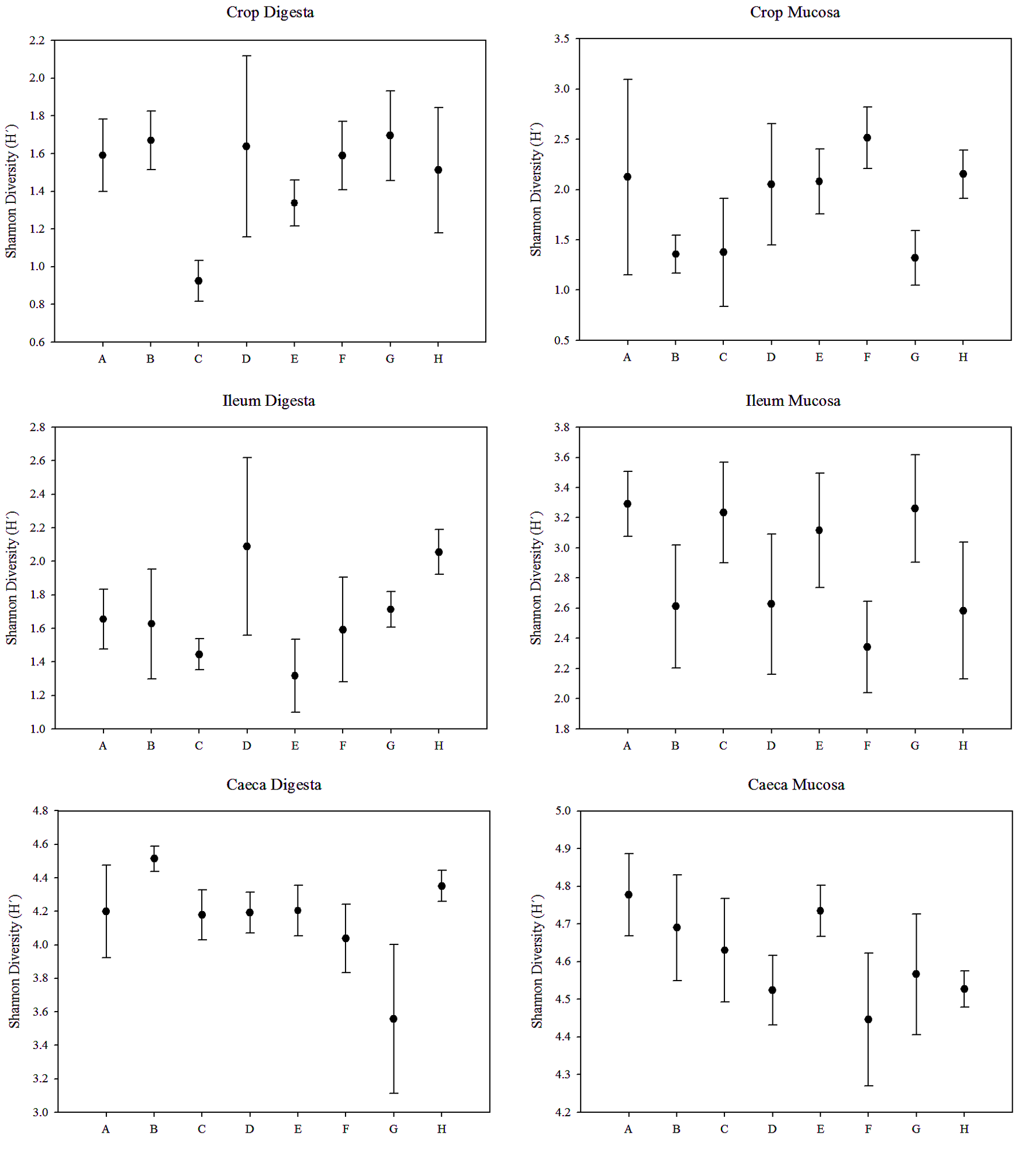

Supplement: Supplementary Figure 4 — Diversity observed across the three GIT sections studied: crop, ileum, and caeca and the two type of samples: digesta and mucosa, for the eight dietary treatments. Values are calculated based on the Shannon diversity index. [file Image4.TIF]
